# Use of Network Pharmacology to Explore the Mechanism of Gegen (*Puerariae lobatae* Radix) in the Treatment of Type 2 Diabetes Mellitus Associated with Hyperlipidemia

**DOI:** 10.1155/2021/6633402

**Published:** 2021-04-16

**Authors:** Guozhen Yuan, Shuai Shi, Qiulei Jia, Jingjing Shi, Shuqing Shi, Xuesong Zhang, Xintian Shou, Xueping Zhu, Yuanhui Hu

**Affiliations:** Department of Cardiology, Guang'anmen Hospital, China Academy of Chinese Medical Sciences, Beijing, China

## Abstract

Rapid increases in metabolic disorders, such as type 2 diabetes mellitus (T2DM) and hyperlipidemia, are becoming a substantial challenge to worldwide public health. Traditional Chinese medicine has a long history and abundant experience in the treatment of diabetes and hyperlipidemia, and *Puerariae lobatae* Radix (known as Gegen in Chinese) is one of the most prevalent Chinese herbs applied to treat these diseases. The underlying mechanism by which Gegen simultaneously treats diabetes and hyperlipidemia, however, has not been clearly elucidated to date. Therefore, we systematically explored the potential mechanism of Gegen in the treatment of T2DM complicated with hyperlipidemia based on network pharmacology. We screened the potential targets of Gegen, T2DM, and hyperlipidemia in several online databases. Then, the hub targets were analyzed by performing protein-protein interaction, Gene Ontology (GO), and Kyoto Encyclopedia of Genes and Genomes (KEGG) enrichment assays, and finally, the complicated connections among compounds, targets, and pathways were visualized in Cytoscape. We found that isoflavones, including daidzein, genistein, and puerarin, as well as *β*-sitosterol, are the key active ingredients of Gegen responsible for its antidiabetic and antihyperlipidemia effects, which mainly target AKR1B1, EGFR, ESR, TNF, NOS3, MAPK3, PPAR, CYP19A1, INS, IL6, and SORD and multiple pathways, such as the PI3K-Akt signaling pathway; the AGE-RAGE signaling pathway in diabetic complications, fluid shear stress, and atherosclerosis; the PPAR signaling pathway; insulin resistance; the HIF-1 signaling pathway; the TNF signaling pathway; and others. These active ingredients also target multiple biological processes, including the regulation of glucose and lipid metabolism, the maintenance of metabolic homeostasis, and anti-inflammatory and antioxidant pathways. In conclusion, Gegen is a promising therapeutic phytomedicine for T2DM with hyperlipidemia that targets multiple proteins, biological processes, and pathways.

## 1. Introduction

The rapidly increasing incidence of chronic metabolic diseases has become a global health threat, among which diabetes mellitus (DM) and hyperlipidemia must be mentioned. First, the latest global estimate from the International Diabetes Federation is that 463 million people suffered from diabetes mellitus in 2019 and that the number will be 700 million by 2045 [1]. At the same time, approximately 90% of cases of type 2 diabetes mellitus (T2DM) are diagnosed [2]. In addition, hyperlipidemia is a well-known risk factor for cardiovascular and cerebrovascular diseases such as atherosclerosis, coronary heart disease, and stroke [3–6]. The morbidity of lipid disorders remains high in both developing and developed countries. In Western Europe and the United States, at least one-third of the population has hypercholesterolemia [7, 8]. In China, the overall prevalence of dyslipidemia in adults was 40.4% in 2012 [9]. Moreover, these two metabolic disorders are both closely related to cardiovascular disease [10]. The majority of patients with diabetes mellitus and/or hyperlipidemia ultimately succumb to heart or blood vessel disease [11, 12].

In fact, the two groups often overlap; namely, someone may suffer from both diabetes and hyperlipidemia. Compared with individuals without dyslipidemia, the risk for diabetes in subjects with hyperlipidemia increases approximately 2- to 3-fold [13]; more than 75% of patients with T2DM have lipid disorders [14]. Diabetes mellitus and lipoproteins are highly interregulated, and thus the complex relationship between diabetes and hyperlipidemia remains unclear [15]. Undoubtedly, once one metabolic disorder occurs, a higher risk of other complications exists [13, 16], and the prevalence of cardiovascular disease is higher in patients with metabolic disorder [17].

Although many drugs have been successfully applied to treat T2DM and hyperlipidemia, safe and effective medicines targeting both diseases at the same time are still lacking. 3-Hydroxy-3-methylglutaryl-coenzyme A reductase inhibitors (also known as statins), the most widely used lipid-lowering drugs in the clinic, have consistently been reported to cause new-onset diabetes mellitus [18]. In addition, the management of complications of these diseases is still a major challenge in clinical practice and a substantial global healthcare burden [19–21].

As an effective supplementary and alternative medicine, traditional Chinese medicine (TCM) has attracted increasing attention. Chinese medicinal herbs are regarded as a rich source for natural drug development. Gegen, the dried root of the leguminous plant *Pueraria lobata* (Willd.) Ohwi or *Pueraria thomsonii* Benth., is a very popular Chinese herb that has been used as a medicine and food. From the perspective of TCM theory, Gegen has the pharmacological functions of clearing heat and promoting the secretion of saliva and body fluid. In clinical practice, Gegen is one of the commonly used herbs for the treatment of metabolic and cardiovascular diseases, such as diabetes mellitus and hyperlipidemia [22, 23]. Some studies on the effects of Gegen-containing formulas (such as Gegen Qinlian Decoction) and Gegen extracts (such as puerarin) on metabolic disturbances were performed [22, 24], but no one has reported the mechanism by which Gegen acts on T2DM complicated with hyperlipidemia to date.

In addition, the rapid development of computer technology enables the identification of the targets and mechanisms of multicomponent natural herbs, accelerating the process of drug development and application because of its low cost and high efficiency [25, 26]. Accordingly, we applied network pharmacology to systematically explore the potential mechanism of Gegen for treating T2DM associated with hyperlipidemia in an attempt to find a novel and beneficial therapy for this increasingly prevalent concurrent metabolic disorder.

## 2. Materials and Methods

### 2.1. Screening the Active Ingredients of Gegen

Active components of Gegen were selected from the Traditional Chinese Medicine Systems Pharmacology (TCMSP) Database (https://tcmspw.com/tcmsp.php) according to oral bioavailability (OB) ≥ 30% and drug-likeness (DL) ≥ 0.18, two parameters of ADME (absorption, distribution, metabolism, and excretion) properties for evaluating compounds. Furthermore, ingredients not meeting the screening criteria but reported as metabolic regulators were also included by performing text mining.

### 2.2. Predicting the Targets of the Compounds

The canonical simplified molecular input line entry specification (SMILES) of each compound was retrieved from the PubChem database (https://pubchem.ncbi.nlm.nih.gov/) containing the chemical structures of small organic molecules and information on their biological activities. Then, targets of active ingredients were searched in Binding DB (http://bindingdb.org/bind/index.jsp), DrugBank (https://go.drugbank.com/), STITCH (http://stitch.embl.de/), and Swiss Targets Prediction (http://www.swisstargetprediction.ch/) according to the SMILES formula. The target prediction algorithms of these databases are mainly based on the structural features of small-molecule ligands, namely, the chemical structure similarity of compounds.

### 2.3. Predicting Targets of Diseases

“Type 2 diabetes mellitus” and “hyperlipidemia” were entered into OMIM (https://www.omim.org/) and GeneCards (https://www.genecards.org/), respectively, to obtain targets of the diseases. The higher the relevance score of the target predicted in GeneCards, the closer the target to the disease. If too many targets are forecasted, those with scores greater than the median score are empirically considered potential targets. Notably, most proteins and genes have multiple names, such as official names and generic names, and thus their names need to be converted uniformly. The protein targets of compounds were checked in UniProt (https://www.uniprot.org/), an online database that collects protein functional information with accurate, consistent, and rich annotations and was represented as the names of the genes encoding them. Next, all gene names were rechecked in the NCBI gene database (https://www.ncbi.nlm.nih.gov/gene) and converted into Entrez IDs and gene symbols. Repeated targets identified due to nonstandard naming were eliminated. The species of the acquired and checked targets was limited to “*Homo sapiens*.”

### 2.4. PPI Network Construction and Module Extraction

A protein-protein interaction (PPI) analysis of targets was performed to explore the relationship among the targets and the biological processes involved. A Venn diagram of targets of Gegen, T2DM, and hyperlipidemia was drawn using an online visualization tool (https://hiplot.com.cn/), and the intersections were regarded as hub genes, namely, the potential targets of Gegen working on type 2 diabetes with hyperlipidemia. The hub genes were analyzed using String (http://string-db.org/, version 11.0), an online database that integrates experiments, databases, and text mining data for PPI prediction and extraction, with the organism restricted to “*Homo sapiens*” and a confidence score >0.4. Isolated nodes were hidden, namely, proteins without any interaction. The result was exported as a “TSV” format file and imported into Cytoscape, which is an open-source software project for visualizing any network of molecular components and interactions to construct a PPI network [27]. However, the interpretation of a PPI network is quite difficult because of its complexity, and therefore a suitable auxiliary network analysis tool is needed. MCODE is a plug-in of Cytoscape for extracting highly interconnected regions of a network called modules or communities, also known as subnetworks [28]. The module or community is considered a cluster of biological functions, more specifically, protein complexes involved in biological processes as a whole or functional module, such as proteins of the same signaling pathway. Combined with a Gene Ontology (GO) enrichment analysis, the key targets and their biological processes of the network can be predicted, making the explanation of the PPI network more convenient and precise [29, 30].

### 2.5. GO and KEGG Pathway Enrichment Analyses

GO and Kyoto Encyclopedia of Genes and Genomes (KEGG) are both common approaches used to find shared functions among genes based on biological ontologies [31]. Briefly, GO annotates genes to biological processes, molecular functions, and cellular components in a directed acyclic graph structure, and KEGG annotates genes to pathways. ClusterProfiler, a useful tool for gene classification and enrichment analysis, and org.Hs.eg.db, a widely used species annotation package, were run in R4.0, an open-source programming environment, with the strict cutoff of *P* values <0.05 for GO and KEGG enrichment [32–34].

### 2.6. Compound-Target-Pathway Network Construction

Cytoscape was used to construct and analyze a three-layer network in order to understand the complex relationships among compounds, targets, and pathways. Taking advantage of another built-in network analyzer [35], the topological parameters of active ingredients, targets, and pathways were calculated, including the degree, betweenness centrality (BC), and closeness centrality (CC), which helped to forecast the main components and core targets of Gegen.

## 3. Results

### 3.1. Active Ingredients of Gegen

Twelve active ingredients of Gegen were finally included based on ADME attributes and text mining. They are shown in Table 1 and include formononetin, daidzein, genistein, and puerarin.

### 3.2. Targets of Compounds

Targets of active ingredients retrieved from Binding DB, DrugBank, STITCH, and Swiss Targets Prediction (only targets with probability >0 included) were merged by deduplication. Ultimately, we obtained 304 targets of the 12 compounds (see Supplementary Material 1 for more details).

### 3.3. Targets of Diseases

Numerous targets of T2DM and hyperlipidemia were retrieved from the GeneCards database. As mentioned above, we empirically excluded some redundant targets based on their relevance score. Then, by merging the targets from the two disease databases, we obtained 2620 targets for T2DM and 706 for hyperlipidemia (see Supplementary Material 2 for more details).

### 3.4. PPI Network and PPI Modules

**Figure 1:**
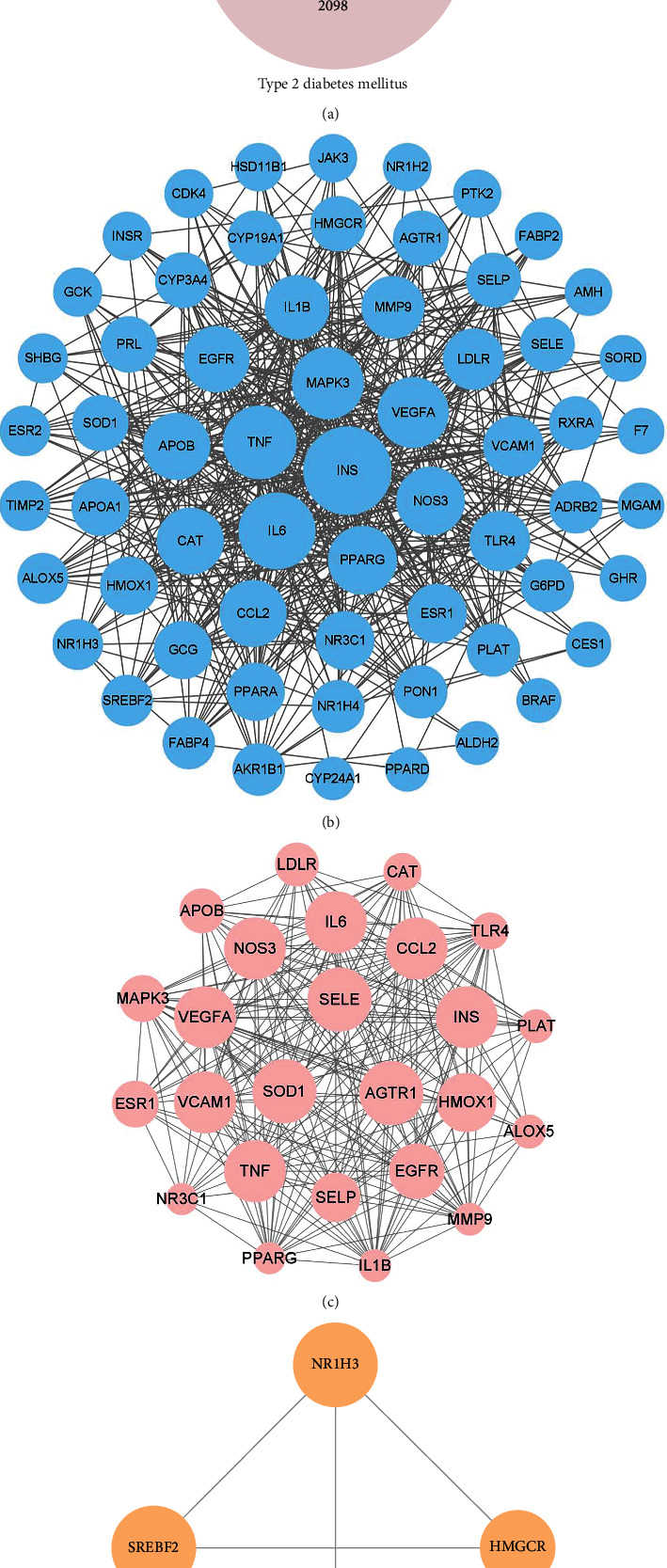
(a) Venn diagram representing the gene targets among Gegen, T2DM, and hyperlipidemia. (b) PPI network of common targets among Gegen, T2DM, and hyperlipidemia, containing 63 nodes and 538 edges. Each node represents a protein produced by a single protein-coding gene locus. An edge represents the interaction between proteins. The greater the number of edges connected to the same node (namely, the greater the degree), the larger the size of the node. (c) Module of the PPI network with the highest score (module 1), containing 25 nodes and 232 edges. (d) Module of the PPI network with the second highest score (module 2), containing 4 nodes and 6 edges. The higher the MCODE score of the node, the larger the size of the node. The MCODE score reflects the density of the node and surrounding nodes. Abbreviations: T2DM, type 2 diabetes mellitus; PPI, protein-protein interaction.

A Venn diagram (Figure 1(a)) was drawn for the targets of Gegen, T2DM, and hyperlipidemia, and 65 common targets were obtained (Table 2). These targets were submitted to STRING 11.0 for the PPI analysis, and the result was visualized using Cytoscape, as shown in Figure 1(b). The PPI network has far more edges than expected, indicating that the proteins are at least partially biologically connected as a group. The entire network is highly interactive. Ins (degree = 57) is the core target in the network, since it interacts with almost all other targets. In addition, high-degree targets are mainly distributed in cholesterol metabolism (PPAR-*γ*, APOB, and LDLR), inflammation (IL6, TNF, VEGFA, NOS3, CCL2, IL1B, and VCAM1), and oxidative stress (MAPK3, NOS3, and CAT).

Modules were extracted from the PPI network using MCODE, and the 2 modules with the highest scores are displayed in Figures 1(c) and 1(d). Combined with the GO enrichment analysis, the primary biological processes in the PPI network and modules were selected according to the false discovery rate to describe their biological functions. The outcomes show that the primary biological process of the PPI network is the same as that of module 1, namely, the response to oxygen-containing compounds (GO: 1901700). The primary biological process of module 2 is the regulation of cholesterol storage (GO: 0010885).

### 3.5. GO Enrichment Analysis of the Targets

**Figure 2:**
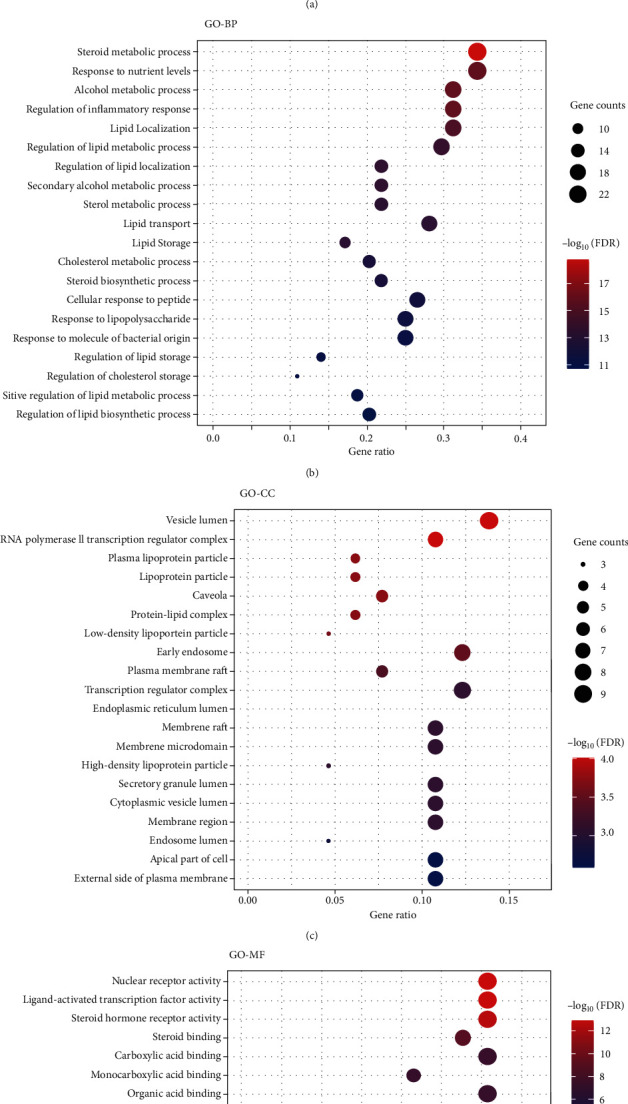
GO enrichment analysis of the 65 common targets of Gegen, T2DM, and hyperlipidemia. (a) The ratio of the GO terms. Biological processes (red), cellular components (yellow), and molecular functions (blue) accounted for 91.60%, 2.78%, and 5.63%, respectively. (b–d) Bubble plots of the top 20 GO terms for biological processes, cellular components, and molecular functions. Abbreviations: BP, biological process; CC, cellular component; MF, molecular function; FDR, false discovery rate, namely, the adjusted *P* value.

Biological process (BP, GO: 0008150), cellular component (CC, GO: 0005575), and molecular function (MF, GO: 0003674) enrichment analyses of 65 common targets were performed using the ClusterProfiler package in R. The top 20 terms significantly enriched in BP, CC, and MF are shown in Figure 2 (*P* < 0.05, *P* values were corrected by the Benjamini-Hochberg procedure). The largest number of BP terms was enriched at 1286. Almost all of the top 20 BPs are involved in the regulation of metabolic processes. In addition, regulation of the inflammatory response is also noteworthy. MF terms are second in number (79) and are mainly related to the activity of various receptors and enzymes, as well as molecular binding. CC terms are minimal (38), and the action sites of gene products are mainly located in various types of vesicles, lumens, membranes, and lipoprotein particles or complexes (see Supplementary Material 3 for more details).

### 3.6. Compound-Target-Pathway Network

**Figure 3:**
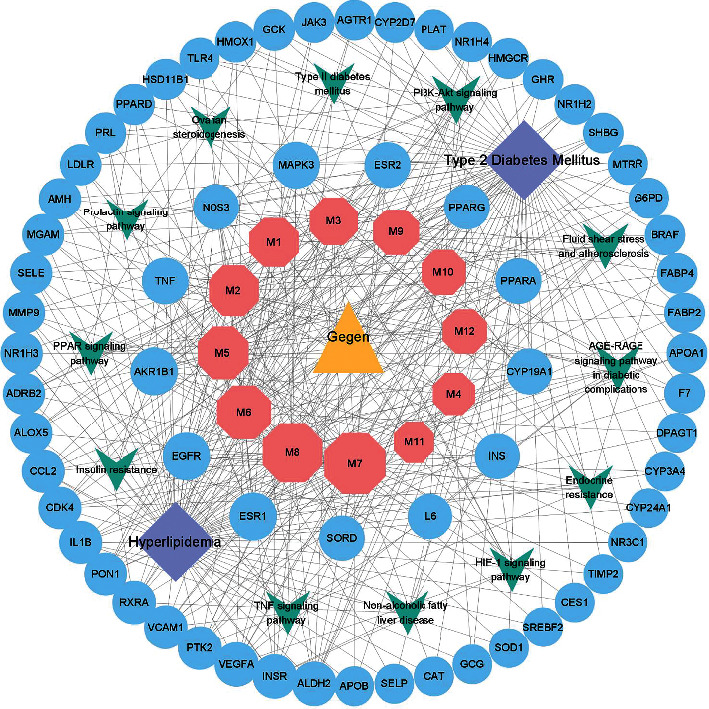
Compound-target-pathway network. The yellow triangle represents the herb, the pink hexagons represent the active ingredients of Gegen, the blue circles represent the common targets between the compounds and the diseases, the purple diamonds represent the diseases, and the green V shapes represent the pathways. Edges represent the interactions among ingredients, targets, and pathways. The greater the number of edges connected to the same node, the larger the size of the node.

The complicated interactions among active components of Gegen, targets, and pathways were visualized with Cytoscape, as shown in Figure 3. By analyzing this three-layer network based on network topology, the degree, BC, and CC of daidzein are 30, 0.0498, and 0.4840, respectively; thus, daidzein is predicted to be the main bioactive component of Gegen in the treatment of T2DM complicated with hyperlipidemia, followed by genistein (degree = 28, BC = 0.0454, and CC = 0.4740), puerarin (degree = 21, BC = 0.0285, and CC = 0.4417), and *β*-sitosterol (degree = 19, BC = 0.0207, and CC = 0.4099). The top three targets with the highest degree value in the network were AKR1B1 (degree = 13, BC = 0.0219, and CC = 0.5000), EGFR (degree = 13, BC = 0.0202, and CC = 0.5000), and ESR1 (degree = 13, BC = 0.0188, and CC = 0.5000), suggesting that they are crucial genes involved in the effect of Gegen on targeting T2DM with hyperlipidemia, and TNF, NOS3, MAPK3, PPAR-*γ*, PPAR-*α*, ESR2, CYP19A1, INS, IL6, and SORD are also relatively important targets (see Supplementary Material 4 for more topological parameters).

Moreover, signaling pathways are an essential part of system pharmacology and associate receptor-ligand interactions with pharmacodynamic outputs. Sixty of all targets were mapped to 96 KEGG pathways (see Supplementary Material 3 for more details). Twelve of the top 20 pathways, according to the adjusted *P* value, are closely related to metabolic disorders. These pathways are the PI3K-Akt signaling pathway (degree = 14), AGE-RAGE signaling pathway (degree = 11), fluid shear stress and atherosclerosis (degree = 11), endocrine resistance (degree = 9), HIF-1 signaling pathway (degree = 9), PPAR signaling pathway (degree = 8), insulin resistance (degree = 8), TNF signaling pathway (degree = 8), nonalcoholic fatty liver disease (degree = 8), prolactin signaling pathway (degree = 6), type II diabetes mellitus (degree = 5), and ovarian steroidogenesis (degree = 5), and much more information is available in Supplementary Material 4.

## 4. Discussion

Currently, the increase in the global prevalence of metabolic diseases represented by T2DM and hyperlipidemia has become an urgent public health problem to solve. T2DM is recognized as a chronic metabolic disorder that affects carbohydrate, lipid, and protein metabolism. A high prevalence of dyslipidemia is a typical feature of T2DM, which has been confirmed in clinical trials [36, 37]. In turn, hyperlipidemia leading to insulin resistance was realized early and strongly proven by clinical trials [38]. Therefore, the two disorders usually coexist because of the complicated connections between them. Undoubtedly, these diseases have imposed tremendous pressure on international public health, especially the prevention and treatment of cardiovascular complications.

As a medication therapy with a long history, traditional Chinese medicine is known for its multitarget synergistic efficacy, based on which homotherapy for heteropathy is put into practice. However, the elucidation of the multitarget effects of TCM is extremely challenging. With the rapid development of computer technology, bioinformatics, proteomics, and network pharmacology have been successfully applied to discover the active ingredients of Chinese herbs and their pharmacological mechanisms. Gegen, a clinically popular Chinese herb, has been prescribed for DM (also known as Xiaoke in TCM) and hyperlipidemia (similar to blood stasis or phlegm retention syndrome in TCM) for many years. In addition, Gegen has the advantage of long-term use with security due to its edibility. Based on these results, Gegen is one of the optimized candidates for diabetic and hyperlipidemic drug development.

In the present study, we screened 12 active components of Gegen and 65 hub targets using network pharmacology. The PPI analysis shows that INS, encoding the insulin protein that plays a vital role in regulating carbohydrate and lipid metabolism, is the gene with the highest degree. Thus, glucose and lipid metabolic disorders are the key to the occurrence of the two diseases discussed, and one of the main benefits of Gegen is to ameliorate this disorder. In addition, cholesterol metabolism, inflammation, and oxidative stress may also be potential mechanisms by which Gegen regulates metabolic disorders. Moreover, module 1 extracted from the PPI network suggests that the excessive accumulation of oxides (GO: 1901700), such as reactive oxygen species, may be one of the common pathological features of metabolic dysfunction, consistent with previous studies that are well summarized in the literature [39]. Module 2 implies that these targets have a significant role in regulating cholesterol metabolism, especially the speed or degree of cholesterol storage (GO: 0010885).

The GO enrichment analysis further revealed that Gegen may regulate lipid metabolism, especially cholesterol metabolism, steroid metabolism, response to nutrient levels, and the inflammatory response through ligand binding, signal transduction, and fatty acid binding in blood, vesicle lumen, caveolae, cell membranes, cytoplasm, and other sites to improve metabolic disorders and exert anti-inflammatory effects. Interestingly, a large number of statistically significant BP terms, such as response to insulin, regulation of insulin secretion, insulin secretion, cellular response to insulin stimulus, glucose metabolic process, glucose homeostasis, and response to glucose, were not at the top of the list (see Supplementary Material 3 for more details). We inferred that the regulatory effect of Gegen on lipids may be more definite than its effect on glucose control from the perspective of evidence-based medicine because the GO annotation and obsoleting are completely based on the latest evidence [40]. Moreover, the benefits of Gegen in treating T2DM and hyperlipidemia are not limited to lowering serum lipid and glucose profiles directly because it also resolves inflammation during the progression of these diseases.

By constructing a three-layer network of compounds, targets, and pathways, we obtained direct insights into the complex interactions among them. We found that these targets are widely involved in insulin resistance and sensitization, glucose and lipid metabolism, inflammation, and diabetes complications. For example, AKR1B1 and SORD have been proven to be associated with the occurrence of complications such as diabetic neuropathy [41, 42]. INS and PPARs are undoubtedly some of the most representative genes regulating metabolism. Insulin plays a vital role in the regulation of saccharides and lipid metabolism. PPAR-*α* is a key regulator of lipid metabolism, such as clearing circulating or cellular lipids. PPAR-*γ* promotes adipocyte differentiation and increases glucose uptake, processes that are essential in the prevention of obesity and the treatment of type 2 diabetes [43].

In addition, EGFR, a gene traditionally considered to regulate cell proliferation and fibrosis, has been shown to be involved in maintaining metabolic homeostasis. Scheving et al. were the first to show that mice with gain-of-function point mutations in the kinase domain of EGFR display elevated plasma and hepatic cholesterol and plasma LDL levels, proving the role of EGFR in regulating lipid metabolism at the basal level [44]. Since then, an increasing number of researchers have verified the role of EGFR in regulating metabolism. Fang et al. observed that EGFR inhibitors were able to reduce inflammation, oxidative stress, fibrosis, and apoptosis in palmitic acid-treated NRK-52E cells and kidneys of high-fat diet-fed mice and improved serum lipid levels and body weight [45]. Estrogen is very important in the metabolic regulation of the whole body, and estrogen receptor (ER) is highly involved in estrogen-mediated modulation of substrate metabolism. ESR1 is critical for the maintenance of whole-body insulin action and protection against tissue inflammation. According to a previous study, ESR1-knockout female mice exhibited significant weight gain, obviously higher fasting blood glucose and lipid levels, elevated levels of circulating and tissue inflammatory markers (PAI-1, MAPK8, and TNF), increased muscular lipid accumulation, impaired glucose tolerance, and insulin resistance, even when they were fed a normal chow diet [46]. In terms of clinical research, some studies have shown that ESR1 and ESR2 gene polymorphisms are associated with lipid levels and insulin sensitivity in adults, despite racial and sex variability [47–49]. TNF-*α* is one of the most important proinflammatory mediators and a key factor in insulin resistance, the evidence for which was well summarized in a classic review [50]. In addition, in healthy humans, TNF-*α* was proven to inhibit whole-body insulin-mediated glucose uptake and signal transduction by suppressing peripheral insulin-stimulated glucose uptake [51]. NOS3 is crucial for the control of arterial pressure and glucose and lipid homeostasis. NOS3−/− mice appeared hypertensive and presented fasting hyperinsulinemia, hyperlipidemia, and lower insulin-stimulated glucose uptake than wild-type mice [52]. Apart from indirectly reducing the risk of cardiovascular and cerebrovascular adverse events by regulating glucose and lipid metabolism, NOS3 also exerts direct effects on vascular protection by synthesizing NO, which is reported to function as an anti-inflammatory agent and antioxidant, including maintaining vascular homeostasis, maintaining the dilation of the vasculature, protecting the intima, and preventing smooth muscle proliferation [53, 54]. Last but not least, approaches targeting MAPK3 (also known as extracellular signal-regulated kinase 1, ERK1) partially protect obese mice from insulin resistance and hepatic steatosis by decreasing adipose tissue inflammation and by increasing muscle glucose uptake [55].

Consistently, the major bioactive components of Gegen have been widely shown to regulate these targets. Thus, the rich isoflavones in Gegen are the dominant active ingredients responsible for the antidiabetes and antihyperlipidemia effects, including daidzein and genistein that are commonly found in soybeans, as well as puerarin, formononetin, and 3′-methoxydaidzein. Isoflavones are phytoestrogens with potent estrogenic activity that have structural similarity with the human female hormone 17-*β*-estradiol. Hence, isoflavones bind to both alpha and beta estrogen receptors and mimic the action of estrogens on target organs. In addition to estrogen-like and/or antiestrogen activity, numerous studies have claimed the functions of genistein and daidzein in the maintenance of metabolic homeostasis and anti-inflammatory and antioxidant activities, thereby exerting many benefits of chemoprevention of metabolic syndrome (MS), obesity, and cardiovascular disease, as well as in relieving postmenopausal symptoms [56–59]. In terms of metabolic regulation, Zucker rats and RAW 264.7 cells treated with a protein mixture or extract of genistein and/or daidzein exhibited antidiabetic effects similar to PPAR agonists, with improved lipid metabolism and activated PPAR receptors [60]. Clinically, a meta-analysis of seventeen randomized controlled trials showed that soy isoflavones significantly improve glucose metabolism in menopausal women [61]. With respect to inhibiting the inflammatory response and oxidative stress, genistein was reported to ameliorate fatty liver in insulin-resistant rats by activating the antioxidant profile, decreasing IL6 and TNF-*α* concentrations and preventing oxidative damage [62]. In addition, apoptosis and proliferation inhibition in human umbilical vein endothelial cells incubated with hydrogen peroxide and high glucose are prevented by genistein and daidzein through the regulation of ESR2 and Bcl-2/Bax expression and modulation of cell survival-related signaling pathways, such as the PI3K pathway [63].

As the most abundant secondary metabolite, puerarin is a unique isoflavone of Gegen. Due to its multiple pharmacological functions, such as vasodilation, cardioprotection, and antioxidant and anti-inflammatory effects, along with the attenuation of insulin resistance, puerarin has been widely used to treat cardiovascular and cerebrovascular diseases, diabetes, and diabetic complications [64], as proven in vivo and in vitro. Puerarin exerts positive hypoglycemic and hypolipidemic roles on mice with diabetes induced by streptozotocin by increasing insulin expression and maintaining metabolic homoeostasis, and it exerts a regulatory effect on lipid accumulation in oleic acid-treated HepG2 cells by decreasing lipogenesis and increasing antioxidant activity, both indicating that puerarin extract has therapeutic benefits in the treatment of glucose- and lipid-related metabolic disorders [65, 66]. Puerarin was able to dose-dependently reduce the phosphorylation of ERK, the expression of TNF-*α* and NOS3, and the release of TNF-*α* and NO to inhibit inflammatory signaling in RAW264.7 macrophages treated with high concentrations of free fatty acids, which are often increased in patients with T2DM and MS [67].

*β*-Sitosterol, a prevalent plant cholesterol derivative (phytosterol) known as the “key of life”, is another pivotal active ingredient in Gegen. As a natural PPAR-*γ* agonist, *β*-sitosterol could be used as a potential therapeutic phytomedicine for the management of metabolic disorders, and overwhelming evidence has been obtained from basic experiments. For example, Gumede et al. discovered that *β*-sitosterol prevents high-fructose diet-induced metabolic dysfunction in female rats, including visceral obesity, hypertriglyceridemia, and hypoadiponectinemia [68]. Another in vivo study showed that the administration of *β*-sitosterol significantly increased the levels of insulin, hemoglobin, and the PPAR-*γ* and GLUT4 proteins, alleviated insulin resistance, and decreased the plasma glucose levels in high-fat diet- and streptozotocin-induced diabetic rats [69]. In addition, a clinical cohort study revealed rather specific negative correlations between the serum sitosterol level and the serum IL6 and TNF-*α* levels in both subjects with and without diabetes, suggesting the anti-inflammatory potential of *β*-sitosterol [70].

Interacting targets form signaling pathways that may reveal the mechanism of a disease. The PI3K-Akt signaling pathway, PPAR signaling pathway, and insulin resistance play pivotal roles in insulin secretion, glucose uptake, and lipid metabolism to maintain glucose and lipid homeostasis [71, 72]. The AGE-RAGE signaling pathway has been proven to exert critical effects on the occurrence and development of diabetic complications, especially vascular complications, and the evidence based on a large number of studies was reviewed elsewhere [73]. Fluid shear stress and atherosclerosis, the HIF-1 signaling pathway, and the TNF signaling pathway are primarily involved in the secretion of proinflammatory factors, matrix degradation, angiogenesis, regulation of vascular tone, leucocyte recruitment, and cell adhesion, which are a series of inflammation-related events that eventually lead to vasculopathy, such as atherosclerosis.

In summary, the main active ingredients of Gegen are isoflavones, such as daidzein, genistein, and puerarin, as well as *β*-sitosterol, a natural triterpenoid. To some extent, these components have consistent multitarget effects. Briefly, the efficacy of Gegen is mainly attributed to regulating insulin secretion and sensitivity, glucose metabolism, and fatty acid and cholesterol synthesis and decomposition to maintain metabolic homeostasis (INS, PPAR-*γ*, PPAR-*α*, and EGFR). On the other hand and more importantly, it also regulates the expression of proteins that mediate complications and risk factors for diabetes and hyperlipidemia, including diabetic cataracts, diabetic retinopathy, diabetic neuropathies, inflammation, atherosclerosis, liver steatosis, and obesity (AKR1B1, SORD, TNF, NOS3, IL6, EGFR, ESR1, and ESR2), to alleviate pathological progression and improve the prognosis. Thus, we speculated that the abundant bioactive ingredients of Gegen may work simultaneously on multiple targets in the PI3K-Akt signaling pathway, AGE-RAGE signaling pathway, fluid shear stress, and atherosclerosis to exert a synergistic therapeutic effect on T2DM and hyperlipidemia, which not only regulates glucose and lipid metabolism directly but also prevents and improves complications secondary to glucose and lipid metabolic disorders. Of course, this study has some limitations. We did not perform in vivo or in vitro experiments to validate the results. We would be willing to supplement the research in the future if the conditions are available and even try to design a clinical trial for verification.

## 5. Conclusions

Taken together, we have discovered that isoflavones and *β*-sitosterol are the critical active components of Gegen responsible for its antidiabetic and antihyperlipidemia effects, which involve multiple mechanisms, such as ameliorating insulin resistance, increasing glucose uptake, promoting adipocyte differentiation, reducing lipid accumulation, and resisting inflammation and oxidative stress. These results are consistent with the main findings of previous studies, providing valuable information for the further development of Gegen. Specifically, Gegen or its bioactive extract has the potential to be applied to the treatment of T2DM and hyperlipidemia independently as a new medicine. After all, Gegen has been used only in the form of a compound prescription (a prescription consisting of 2 or more different Chinese medicines) for clinical treatment to date. Compared with Chinese herbal compounds, the mechanism of action of single Chinese herbs and monomeric compounds is easier to explain and apply in the clinic. Overall, Gegen is a safe, effective, and promising natural phytomedicine for the treatment of type 2 diabetes accompanied by hyperlipidemia with the advantages of multiple ingredients, targets, biological processes, and pathways.

## Figures and Tables

**Table 1:** Active ingredients and ADME parameters of Gegen.

No.	MOL ID	Molecule name	OB (%)	DL
M1 ^*∗*^	MOL001999	Scoparone	74.75	0.09
M2	MOL000392	Formononetin	69.67	0.21
M3	MOL002959	3′-Methoxydaidzein	48.57	0.24
M4	MOL003629	Daidzein-4,7-diglucoside	47.27	0.67
M5	MOL000358	Beta-sitosterol	36.91	0.75
M6 ^*∗*^	MOL012297	Puerarin	24.03	0.69
M7 ^*∗*^	MOL000390	Daidzein	19.44	0.19
M8 ^*∗*^	MOL000481	Genistein	17.93	0.21
M9 ^*∗*^	MOL000663	Lignoceric acid	14.9	0.33
M10 ^*∗*^	MOL009720	Daidzin	14.32	0.73
M11 ^*∗*^	MOL000441	Lupenone	11.66	0.78
M12 ^*∗*^	MOL000391	Ononin	11.52	0.78

Notes:  ^*∗*^The compounds do not meet the inclusion criteria based on ADME (OB ≥ 30% and DL ≥ 0.18) but have been reported to have metabolic regulatory effects. Abbreviations: OB, oral bioavailability; DL, drug-likeness.

**Table 2:** Common targets of Gegen, type 2 diabetes mellitus, and hyperlipidemia.

Entrez ID	Gene symbol	Uniprot ID	Protein name
154	ADRB2	P07550	Beta-2 adrenergic receptor
185	AGTR1	P30556	Type-1 angiotensin II receptor
231	AKR1B1	P15121	Aldo-keto reductase family 1 member B1
217	ALDH2	P05091	Aldehyde dehydrogenase, mitochondrial
240	ALOX5	P09917	Polyunsaturated fatty acid 5-lipoxygenase
268	AMH	P03971	Muellerian-inhibiting factor
335	APOA1	P02647	Apolipoprotein A-I
338	APOB	P04114	Apolipoprotein B-100
673	BRAF	P15056	Serine/threonine-protein kinase B-raf
847	CAT	P04040	Catalase
6347	CCL2	P13500	C-C motif chemokine 2
1019	CDK4	P11802	Cyclin-dependent kinase 4
1066	CES1	P23141	Liver carboxylesterase 1
1588	CYP19A1	P11511	Aromatase
1591	CYP24A1	Q07973	1,25-Dihydroxyvitamin D(3) 24-hydroxylase, mitochondrial
1564	CYP2D7	A0A087X1C5	Putative cytochrome P450 2D7
1576	CYP3A4	P08684	Cytochrome P450 3A4
1798	DPAGT1	Q9H3H5	UDP-N-acetylglucosamine-dolichyl-phosphate N-acetylglucosaminephosphotransferase
1956	EGFR	P00533	Epidermal growth factor receptor
2099	ESR1	P03372	Estrogen receptor
2100	ESR2	Q92731	Estrogen receptor beta
2155	F7	P08709	Coagulation factor VII
2169	FABP2	P12104	Fatty acid-binding protein, intestinal
2167	FABP4	P15090	Fatty acid-binding protein, adipocyte
2539	G6PD	P11413	Glucose-6-phosphate 1-dehydrogenase
2641	GCG	P01275	Pro-glucagon
2645	GCK	P35557	Hexokinase-4
2690	GHR	P10912	Growth hormone receptor
3156	HMGCR	P04035	3-Hydroxy-3-methylglutaryl-Coenzyme A reductase
3162	HMOX1	P09601	Heme oxygenase 1
3290	HSD11B1	P28845	Corticosteroid 11-beta-dehydrogenase isozyme 1
3553	IL1B	P01584	Interleukin-1 beta
3569	IL6	P05231	Interleukin-6
3630	INS	P01308	Insulin
3643	INSR	P06213	Insulin receptor
3718	JAK3	P52333	Tyrosine-protein kinase JAK3
3949	LDLR	P01130	Low-density lipoprotein receptor
5595	MAPK3	P27361	Mitogen-activated protein kinase 3
8972	MGAM	O43451	Maltase-glucoamylase, intestinal
4318	MMP9	P14780	Matrix metalloproteinase-9
4552	MTRR	Q9UBK8	Methionine synthase reductase
4846	NOS3	P29474	Nitric oxide synthase, endothelial
7376	NR1H2	P55055	Oxysterols receptor LXR-beta
10062	NR1H3	Q13133	Oxysterols receptor LXR-alpha
9971	NR1H4	Q96RI1	Bile acid receptor
2908	NR3C1	P04150	Glucocorticoid receptor
5327	PLAT	P00750	Tissue-type plasminogen activator
5444	PON1	P27169	Serum paraoxonase/arylesterase 1
5465	PPARA	Q07869	Peroxisome proliferator-activated receptor alpha
5467	PPARD	Q03181	Peroxisome proliferator-activated receptor delta
5468	PPARG	P37231	Peroxisome proliferator-activated receptor gamma
5617	PRL	P01236	Prolactin
5747	PTK2	Q05397	Focal adhesion kinase 1
6256	RXRA	P19793	Retinoic acid receptor RXR-alpha
6401	SELE	P16581	E-selectin
6403	SELP	P16109	P-selectin
6462	SHBG	P04278	Sex hormone-binding globulin
6647	SOD1	P00441	Superoxide dismutase [Cu-Zn]
6652	SORD	Q00796	Sorbitol dehydrogenase
6721	SREBF2	Q12772	Sterol regulatory element-binding protein 2
7077	TIMP2	P16035	Metalloproteinase inhibitor 2
7099	TLR4	O00206	Toll-like receptor 4
7124	TNF	P01375	Tumor necrosis factor
7412	VCAM1	P19320	Vascular cell adhesion protein 1
7422	VEGFA	P15692	Vascular endothelial growth factor A

## Data Availability

The data used to support the findings of this study are included within the supplementary information files.

## Supplementary Materials

Supplementary Materials Supplementary materials include the following. Supplementary Material 1: active ingredients and targets of Gegen. Supplementary Material 2: targets of type 2 diabetes and hyperlipidemia. Supplementary Material 3: results of GO and KEGG enrichment. Supplementary Material 4: topological parameters of the compound-target-pathway network.
